# HIV-Associated Venous Thromboembolism

**DOI:** 10.4084/MJHID.2011.030

**Published:** 2011-07-08

**Authors:** Michele Bibas, Gianluigi Biava, Andrea Antinori.

**Affiliations:** Clinical Department, National Institute for Infectious Diseases “Lazzaro Spallanzani”, IRCCS, Rome, Italy

## Abstract

HIV infection has been recognized as a prothrombotic condition and this association has now been proven by a large number of studies with a reported VTE frequency among HIV-infected patients ranging from 0.19% to 7,63 %/year. HIV infection is associated with a two to tenfold increased risk of venous thrombosis in comparison with a general population of the same age. Some risk factors demonstrated a strongest association with VTE such as, low CD4^+^ cell count especially in the presence of clinical AIDS, protein S deficiency, and protein C deficiency. Whereas other risk factors are still controversial like protease inhibitor therapy, presence of active opportunistic infections and presence of antiphospholipid antibodies, including anticardiolipin antibodies and lupus anticoagulant. Physicians caring for HIV positive patients should be able to recognize and treat not only the well-known opportunistic infections and malignancies associated with this chronic disease, but also be alert to the less well-known complications such as thromboses. Pulmonary embolism should be included in the differential diagnosis when patients with HIV/AIDS have unexplained dyspnea or hypoxemia. In younger individuals with VTE, especially men, without other identifiable risk factors for VTE, HIV should be considered. Because interactions between warfarin and antiretrovirals is possible, health care providers should also be alert to the potential of dangerously high or low INRs when they are giving anticoagulants to patients with HIV infection who are undergoing antiretroviral therapy.

## Introduction:

Human immunodeficiency virus (HIV) infection results from one of two similar retroviruses (HIV-1 and HIV-2) that destroy CD4+ lymphocytes and impair cell-mediated immunity, affecting multiple organ systems. HIV manifestation ranges from asymptomatic carriage to the acquired immune deficiency syndrome (AIDS), which is defined by serious opportunistic infections or cancers. In 2009, there were worldwide an estimated 33.3 million (31.4 million – 35.3 million) of adults and children living with HIV, 2.6 million [2.3 million–2.8 million] people became newly infected with HIV, and 1.8 million (1.6–2.1 million) are the AIDS-related death among adults and children.^1^ Treatment with highly active antiretroviral therapy (HAART) has successfully prolonged the life expectancy of HIV-infected patients and infection with the human immunodeficiency virus is increasingly becoming a chronic disease in the developed world.^2^.^3^ Improved survival has been followed by an increased and anticipated prevalence of non-AIDS related conditions, in particular cardiovascular disease is now a leading cause of morbidity and mortality among HIV-infected people.^4^

## Epidemiology:

Venous thromboembolism (VTE) is a common, serious disease with an estimated incidence rate in the general population of 1 per 1000 person-years of observation.^5^ Prevention and treatment of VTE are gaining attention because of an increase in frequency, cost, and risk factors. Furthermore VTE is a potentially preventable disease and it is of utmost importance to identify individuals in high-risk populations who may benefit from primary thromboprophylaxis.^6^,^7^ HIV infection has been recognized as a prothrombotic condition and this association has now been proven by a large number of studies. In fact many epidemiological studies reported on the occurrence of VTE among HIV-infected patients with a frequency ranging from 0.19 to 7,63 %/year.^8^–^20^ These studies (Table 1) estimates that the overall increase of the risk of VTE in HIV-infected patients was 2–10-fold higher than expected in general population. However many trials were limited by small sample size and a lack of a population based comparison control, and mainly were conducted in the pre-HAART era. Few studies were conducted in the more recent HAART era.^8^–^20^ Of interest recently Rasmussen found that the 5-year risk of VTE was 8.0% in injecting drug users (IDU) HIV-infected patients, 1,5% in non-IDU HIV-infected patients and 0.3 % in the population comparison cohort.^20^

Although HIV-infected patients are at increased risk for venous thromboembolism little work has been done on defining the exact mechanisms by which this phenomenon occurs, and still less has been done on evaluating the role thromboprophylaxis in HIV-infected individuals. Notably the 2008 *American College of Chest Physicians* (ACCP) guidelines on antithrombotic and thrombolytic therapy are silent on this subject.^7^ Furthermore there are some important concerns about the therapy of HIV-related thromboses. The aim of this review is to provide an overview about the venous thromboembolism in HIV-infected individuals, trying to cover pathogenesis, prophylaxis and treatment issues.

## Risk Factors for Thromboembolism in Hiv-Infected Patients:

**Figure 1:**
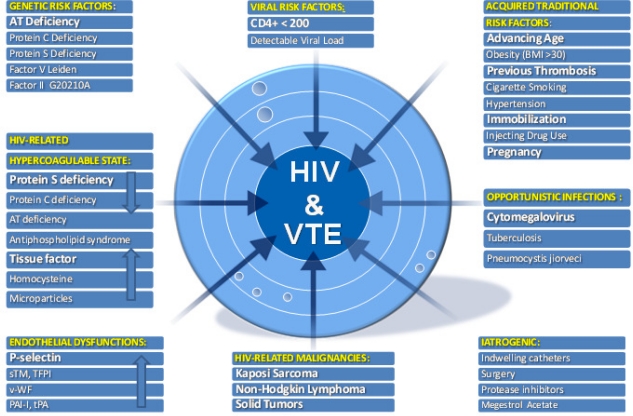
Multi-factorial etiology of HIV-related venous thromboembolism. AT, antithrombin; sTM, soluble thrombomodulin; TFPI, tissue factor pathway inhibitor; v-WF, von Willebrand Factor; PAI-I, plasminogen activator inhibitor-1; tPA, tissue plasminogen activator. Stronger risk factors for VTE are listed in bold.

VTE is a multicausal disease and most commonly is the result of more than one “Hit”. The probability of developing venous thromboses would depend on type and number of risk factors involved (Figure 1). Many established factors are known to increase the risk of VTE in general population.^21^ Furthermore several specific factors are thought to be associated with VTE in patients with HIV. For convenience we grouped them in three categories: those regarding the host, mainly defining a hypercoaglulable state and endothelial dysfunctions, those regarding the HIV diseases state, and those regarding the therapy whether HAART or other.

## Host Risk Factors:

### Age:

In the developed world advancing age is a well known risk for thrombosis in general population. The incidence of venous thromboembolism increase dramatically as the population ages, from 0,001% a year in childhood to nearly 1% a year in the elderly.^22^ Because most HIV infected people are relatively young, their risk of DVT should be expected to be lower than the overall incidence. Conversely many studies reported that HIV-infected patients had a median age of 40 at time of venous thromboembolism that is 20 years younger than the median age of non infected patients.^12^,^15^ Furthermore patients younger than 50 years with HIV had a significantly higher incidence rate of VTE/year compared with age-matched healthy controls.^23^ HIV-infected patient are in fact older than their chronological age and they experience the so-called “Premature Aging”. In this immunological ageing the immune system has persistent defects even after years of treatment mediated viral suppression. Many are similar to those seen in normal ageing, but they occur at an earlier age than normal.^24^ Persistent abnormalities include low CD4:CD8 ratio, low naïve:memory cell ratio, expansion of CD28− effector T cells, reduced T cell repertoire, and reduced responsiveness to vaccines. Most of these abnormalities are seen only in patients who start treatment in late stage disease (CD4 nadir <200 cells).^25^ The heightened risk for premature aging is also the result of residual immunodeficiency and inflammation.^26^

### Intravenous Drug Use:

The intravenous use of recreational drugs is associated with considerable morbidity, a significant proportion of which may be from the drug itself.^27^ Intravenous drug use has been recently identified as an important cause of community-acquired VTE in young adults.^28^ In a recent elegant paper Rasmussen first show the impact of intravenous drug use on risk Of VTE in HIV-infected patients.^20^ This study found that the risk of VTE was nearly 15 times higher in IDU HIV-infected patients that in non IDU HIV-infected patients.

### Hypercoagulable State:

#### Protein S Deficiency:

It is currently accepted that protein S (PS) deficiency is a significant contributor to the pro-coagulant nature of HIV infection. In fact protein S deficiency is the most consistently observed coagulation abnormality observed in HIV-infected patients, with a reported prevalence ranging from a 27% to a 76%, with a 12% of those patients having a venous thromboembolism.^29^–^32^ PS deficiency in HIV-positive patients is probably multi-factorial and the frequency of this alteration/deficiency has led to an extensive pursuit of potential mechanism. Type III PS deficiency is the most common abnormality found and characterized by a normal total protein S level with a decrease in both free protein S and functional protein S activity.^33^ Decreased synthesis by the endothelial cells, hepatocytes and megakariocytes injured in HIV infection has been proposed.^30^ A positive correlation between antibodies to PS and low levels of circulating free antigen has also been noted in symptomatic HIV-infected patients.^34^ Other authors suggested that tumor necrosis-factor-alpha (TNF-α) can lower the levels of active protein S down-regulating the protein S synthesis in the endothelial cells.^35^ Another publication showed how the occurrence of PS deficiency might be linked to the presence of antiphospolipid antibodies.^36^

#### Protein C Deficiency:

The relationship between protein C deficiency and VTE in HIV infection is not as clear as that of protein S deficiency, with a prevalence ranging from 0 to 14%. However in one study a high prevalence of protein C deficiency was found in HIV patients who had VTE without other noted risk factors for venous thromboembolism.^31^ The mechanism of PC deficiency in HIV infected persons is multi-factorial, including altered synthesis and metabolism as well as low-grade disseminated intravascular coagulation (DIC) with consumptive coagulopathy.^37^

#### Antithrombin Deficiency:

There is no direct evidence of HIV infection leading to Antithrombin deficiency. Acquired AT deficiency frequently occurs in the course of HIV disease as a consequence of associated conditions that cause decreased protein synthesis (liver diseases eg HCV coinfections and malnutrition), protein-losing nephropathies or enteropathies, consumptive states (malignancy, DIC, surgery). Nevertheless cases of AT deficiency in HIV patients who experienced thrombotic events have been reported.^14^,^38^,^39^

#### Antiphospholipid And Lupus Anticoagulant Antibodies:

The antiphospholipid syndrome (APS) is an autoimmune disease associated to the appearance of two main circulating auto-antibodies, anticardiolipin antibodies (ACA) and lupus anticoagulant (LA). The frequency of APS in the general population is 2–4%, and is clearly linked to increased risk of venous and arterial thrombosis.^40^ ACA have been reported in HIV-infected patients with a prevalence ranging from 7% to 94%.^41^ However there are both reports of no association^42^,^43^ and positive association between having positive ACA and VTE in HIV+ patients.^44^,^45^ LA is much more variable in frequency and manifestation in HIV+ patients. The incidence ranging from 0% to 72% but no pathogenic correlation was found with thromboses in two large series of HIV patients[]^44^,^45^ even if some case reports are found in the literature.^46^ Actually is thought that LA activity in those patients might be an epiphenomenon secondary to chronic immune stimulation in HIV infection.

#### Tissue Factor:

Recently Funderburg et al^47^ found dramatically higher frequencies of monocytes expressing tissue factor (TF) in fresh blood samples from HIV-infected persons than in samples from uninfected controls. They postulated that a variety of bacterial toll-like receptor (TLR) ligands, such as peptidoglycans, lipopolysaccharide (LPS), and flagellins, are translocated through the damaged gut in chronic HIV infection and may drive immune activation (in addition to HIV viral Replication) and monocyte TF expression in this setting.^48^ The relevance of increased TF expression in HIV infection is underscored by the high levels of D-dimers in plasma and by the correlation between TF expression and D-dimer levels. HIV replication and systemic translocation of microbial products from the damaged gut, and the subsequent immune activation, contribute to a procoagulant state in HIV-infected patients that is due, at least in part, to increased surface expression of TF on circulating monocytes.^48^

#### Microparticles:

The term “microparticles” (MP) refers to a small (< 1mμ) membrane vesicles released from activated or apoptotic cells.^49^ The MPs may be generated from endothelial cells, vascular smooth muscle cells, platelets, tumor cells and from apoptotic CD4+ lymphocytes.^50^ In healthy individuals, very low levels of MPs are present in platelet-free plasma, conversely elevated levels have been identified in HIV-positive patients, but there is no clear evidence that this causes a rise in the risk of VTE.^51^

#### Homocysteine:

Mild to moderate Hyperhomocysteinemia (HHcy) is relatively common in HIV-infected individuals, especially those using cART, with a prevalence ranging from 11 to 29%.^52^,^53^ Prospective cohort studies and an interventional trial in general population have estimated the increase in the risk of recurrence associated with raised homocysteine concentrations to be about 1 to 5-fold.^54^,^55^ However, because vitamin supplementation (which reduces homocysteine concentrations) does not affect rate of recurrence, a causal relation between hyperhomocysteinemia and venous thrombosis remain uncertain.^56^ HHcy is frequently present in HIV-patients without causing clinical manifestation suggesting that it may not be sufficient alone causing VTEs. However HHcy may add an additional risk among patients with other risk factors for venous clots.

#### Endothelial Dysfunction:

Many studies showed a strong association between endothelial cells abnormalities and VTE in general population.^57^–^59^ Under normal conditions endothelial cells exert a vasodilatory, antiplatelet and local fibrinolytic tone that prevents platelet adhesion, leukocyte attachment, as well as blood coagulation.

A non-thrombogenic endothelial surface is maintained through a number of mechanisms, including the production of thrombomodulin (TM) (an activator of anticoagulant protein C), the expression of heparan and dermatan sulphate (which accelerate the thrombin-inhibitory activity of antithrombin III and of heparin cofactor II), the constitutive expression of tissue factor pathway inhibitor (TFPI)(an inhibitor of tissue factor), and the local production of tissue plasminogen activator (tPA) and urokinase-type plasminogen activator (uPA), that are the main effectors of physiologic fibrinolysis. Crucial to many of the antithrombotic activities of endothelium are the synthesis of prostacyclin (PGI2) and of nitric oxide (NO).[60] In the context of VTE, a dysfunctional venous endothelium may express increased amounts of P-selectin, von Willebrand factor (vWF), tissue factor (TF), plasminogen activator inhibitor-1 (PAI-1), and factor V, all of which may promote blood clotting and participate in the development of a thrombus.^61^ It is now well estabilished that the endothelium could be activated directly by HIV virus. In fact multiples studies reported the role of HIV in causing endothelial dysfunction.^62^,^63^

#### P-Selectin:

In a recent study P-selectin was found in HIV-infected patients to be independently and most strongly associated with venous thrombosis.^64^ Stored in endothelial cells and platelet granules, P-selectin interacts with its receptor to promote a hypercoagulable environment by inducing the generation of prothrombotic microparticles from leukocytes and upregulation of tissue factor expression on monocytes.^65^ Prospective studies in HIV-uninfected participants with malignancies have demonstrated that P-selectin is significantly elevated in patients with an impending or acute VTE. Furthermore, P-selectin has been shown to have comparable diagnostic value to D-dimer in patients with confirmed DVTs.^66^

#### Miscellaneous Factors of Haemostasis:

Various markers of endothelial cell damage such as von Willebrand factor (vWF), soluble thrombomodulin (sTM), adhesion molecule E-selectin, tissue-type plasminogen activator (tPA), plasminogen activator inhibitor (PAI-1), fibronectin, angiotensin-converting enzyme (ACE), and endothelin have been shown to be increased in the course of HIV-1 infection.^67^,^68^ HC II deficiency was significantly more pronounced in AIDS patients compared with HIV patients and possible reason for HC II deficiency could be the decreased synthesis, enhanced proteolysis or consumption.[69] However no paper has shown a direct correlation between the low levels of HC II in HIV-positive patients and VTE. The secretion of tPA, (PAI-1), sTM, and vWF creates alterations in the coagulation cascade and could predispose to thrombosis.^70^–^73^ Furthermore, HIV gp120 could induce tissue factor expression in vascular SMCs, which may have potential effects on the arterial wall thrombogenicity.^74^ Anyway a link between in vitro findings and clinical events in HIV patients is still lacking.

## Viral Risk Factors:

### CD4+ Cell Count:

The severity of the HIV infection appears to be of significance in association with VTE. In fact several studies confirmed that there is a higher incidence of venous thrombosis in patients with low CD4 counts.^12^,^37^,^75^,^76^ In particular although the CD4 nadir and most recent CD4 count were both predictive in the univariate models, the strongest predictor in multivariate models was the CD4 cell count at the time of the VTE.^14^,^19^,^77^,^78^ Although the frequency of VTE is higher in the presence of lower CD4^+^ cell counts, there are reports of thrombosis occurring with CD4^+^ cell counts as high as 800 cells/mm^3^, suggesting that the risk of thrombosis is not completely confined to patients with end-stage disease.^19^ The correlation between CD4 count and the risk for the development of thromboses may be related to an increasing hypercoagulable state found with progressive immune suppression and HIV disease progression. Various studies have documented that the abnormalities in pro- and anticoagulant factors, and as described previously, worsens as the disease progresses, swinging the pendulum in favor of thrombosis.^53^,^79^,^80^

### Viral Load:

Another indicator of high disease burden of HIV infection is viral load, also known as HIV RNA level. Low CD4^+^ cell counts and high viral loads are predictive of progression of HIV and typically complement each other in the absence of treatment. One group of authors concluded that a higher viral load, and lower CD4^+^ cell count, was associated with a higher risk of thrombosis,^19^ conversely others found no correlations.^14^

### Opportunistic Infection:

In spite of the efficacy of HAART, HIV-positive individuals are at the greatest risk for developing opportunist infections depending to their immunologic status. So the concomitant presence of advanced HIV disease and opportunistic infections appears to be an additional risk factor for Thrombosis.^8^,^13^,^14^,^76^,^79^

VTE is most commonly reported with Cytomegalovirus and *Pneumocystis jiroveci* pneumonia (PCP) and Mycobacteriumavium-intracellulare.^79^,^80^

### Cytomegalovirus:

Cytomegalovirus active infection is a well-established cause of thrombosis and several case-studies described active cytomegalovirus infection in patient with VTE in general population.^81^–^86^ Regarding HIV-infected patients active cytomegalovirus infection has decreased substantially, in current HAART era, to less than 6 cases/100 person-years.^87^ In one study it was associated with a procoagulant state independently of stage of HIV disease.^88^ The reported frequency of VTE in the presence of cytomegalovirus in HIV-positive individuals is approximately 9.8%, with the majority of thrombosis associated with gastrointestinal-related disease.^12^

### Pneumocystis Jiroveci Pneumonia:

Many authors reported the presence of active PCP during a VTE in HIV-infected patients.^10^,^14^,^23^,^79^ VTE associated with PCP may be secondary to the hypercoagulable state in patients with AIDS. In fact *Pneumocystis jiroveci* pneumonia patients with HIV have been shown to have an high rate of concurrent antiphospholipid syndrome that may increase the risk for developing VTE.^89^ Venous thromboembolism, specifically pulmonary embolism, in this population may remain underdiagnosed because of similar signs and symptoms of presentation between pulmonary embolism and PCP.^90^

### Mycobacterium Tuberculosis and Intracellular mycobacterium Avium:

Mycobacterium-avium-intracellulare and Mycobacterium tuberculosis may induce anticardiolipin antibodies and a hypercoagulable state. Although anticardiolipin antibodies are found in these patients, a clear relationship with the appearance of thromboembolic complications was not demonstrated.^91^ Declining levels of anticardiolipin antibodies seem to occur after the initiation of effective treatment of underlying infection.^47^,^92^ *Mycobacterium tuberculosis* is able to activate macrophages directly and induces them to produce cytokines, especially TNF-α, IL-1 and IL-6. TNF-α and IL-1 blocks the protein C anticoagulant pathway and can elicit tissue factor production on endothelium and monocytes.^93^ IL-6 can also stimulate new platelets formation which have increased sensitivity to thrombin activation and increased pro-coagulant activity. The prevalence of VTE in Tuberculosis patients is ranging from 0.6% to 3%, while the prevalence of VTE in patients with coexisting HIV and tuberculosis is unknown.^94^,^95^

### HIV-associated Malignancy:

The risk of VTE in patients with cancer varies considerably between patients and even within an individual patient over time. Estimates ranging from 15 to 30 % have been reported.^96^ It is well-known how patients with no identifiable risk factors who develops DVT may have an underlying occult malignancy.^97^ People with HIV infection and AIDS have an elevated cancer risk.^98^ Compared with the general population, HIV-infected individuals have a 3640-fold increased risk of Kaposi sarcoma (KS), a 77-fold increased risk of non-Hodgkin lymphomas (NHL), and a six-fold increased risk of cervical cancer.^99^ These malignancies are AIDS-defining cancers, based on the Centers for Disease Control and Prevention (CDC) definition of AIDS.^100^ HIV-infected people also have an increased risk of a number of non-AIDS-defining cancers, including some associated with co-infections (eg, anal and oropharyngeal cancers associated with HPV infection, liver cancer associated with infection with hepatitis B and C viruses, and Hodgkin lymphoma associated with Epstein–Barr virus infection).^101^ Kaposi Sarcoma (KS) is the most common malignancy reported in literature associated with VTE in HIV-patients. Several reviews regarding thromboembolism in patients with KS in HIV-infected patients have reported an incidence of thrombosis ranging from 9.3% to 20%.^13^,^23^,^102^ The remaining malignancies were reported as single cases of primary CNS Lymphoma, B-cell non-Hodgkin lymphoma, Hodgkin disease, prostate cancer, anal cancer, colon cancer.^13^,^19^,^23^

## Drugs Risk Factors:

### HAART:

HAART and in particular the use of protease inhibitors (PI) have been associated with thrombotic events.^11^,^12^,^31^,^32^ PI are thought to interfere with hepatic metabolism, specifically cytochrome P450 metabolism, and regulation of thrombotic proteins. This may ultimately cause a prothrombotic state in HIV-infected individuals and therefore increase the risk of a thrombotic episode. Otherwise they may either downregulate the anticoagulant effect within the body or generate endothelial or platelet dysfunction.^14^ Consistent data exist connecting protease inhibitors with lipodystrophy, and HIV-positive individuals with fat redistribution may be at increased risk for developing an abnormal coagulation profile, such as increased fibrinogen, D-dimer, plasminogen activator inhibitor-1, or protein S deficiency.^103^ Despite limited data, protease inhibitors indinavir and saquinavir, have been associated with an increased risk of VTE in the HIV-positive population,^11^,^23^,^104^,^105^ however a recent publication could prove no such association, suggesting that the association between HAART and risk of thrombosis may arise from mutual associations with other risk factors, in particular advanced disease.^19^

### Megestrol:

Megestrol acetate is a synthetic, orally active, progestational agent, used widely in the treatment of metastasic breast cancer. It has also been reported to stimulate appetite and weight gain in patients with AIDS-related anorexia and/or cachexia. In these groups of patients thromboembolic phenomena as adverse events potentially related to megestrol have been reported.^106^,^107^

## Clinical Presentation:

Clinicians should be alert to unprovoked thrombosis as a possible complication of AIDS and consider this in the differential diagnosis of patients with HIV infection. It is possible that the incidence of thromboembolic disease in those patients have been underestimated either due to the clinical picture mimicking opportunistic infections (e.g., opportunistic pulmonary infection vs. PE) or not considering the less well-known complication such as venous thromboembolism.^108^ The clinical appearance and distribution of thrombosis reported in the literature is similar to non HIV-patients.^14^,^19^,^23^ Most commonly thromboses involve the popliteal and femoral veins followed by pulmonary emboli.[10,36] In addition, abdominal involvement may occur as portal or splenic vein thromboses.^109^,^110^ HIV-infected persons may experience recurrences with an incidence of recurrence rate ranging from 8% to 15 %.^12^

### Pregnancy:

Venous thromboembolism (VTE), in women during pregnancy and puerperium, has been described in literature with an incidence of approximately 1–2 in 1000 pregnancies. Women are five times more likely to develop VTE during pregnancy or puerperium compared to non-pregnant women. A recent study reported the annual incidence of VTE in HIV-positive women during puerperium of 313/1000 person-years (95% CI 65–915).^111^ According to this finding, HIV-positive pregnant women are 120-fold more likely to develop VTE than HIV-positive controls,^12^ whereas the risk is 157-fold higher compared to HIV-negative pregnant women.^111^

## Management:

The 2008 ACCP guidelines on antithrombotic and thrombolytic therapy do not mention HIV-infected patients. Nevertheless clinicians dealing with HIV must be aware that this high risk population needs particular attentions regarding antithrombotic prophylaxis and therapy looking at them in the same way as it would be for patients suffering from cancer. The management of proven VTE in HIV-infected patients should be the same as for the non HIV-patients, including long-term prophylaxis with low molecular weight heparin and warfarin for patients with recurrent thrombosis.

### Antiretroviral therapy and warfarin drug interaction:

Because of an increased risk of thromboembolism in patients with HIV/AIDS and the increasing longevity of the HIV-infected population receiving effective antiretroviral therapy, more HIV-positive patients will be receiving in the future concomitant oral anticoagulant and antiretroviral therapy.

The interaction between antiretroviral agents and other medications has been widely described.^112^ Interactions between warfarin and antiretrovirals is possible, given the influence of many antiretrovirals on CYP2C9 the enzyme responsible for the metabolism of the more active S-enantiomer of warfarin.^113^

Among the antiretrovirals, interactions involving non-nucleoside reverse transcriptase inhibitors (NNRTIs) and protease inhibitors (PIs) with warfarin are most likely to occur. Inhibition or induction of metabolism requiring warfarin dosage adjustment may be observed, depending on the specific antiretroviral drug. Among the NNRTIs, induction of warfarin metabolism is likely with nevirapine. Inhibition of warfarin metabolism may occur with efavirenz, delavirdine, or etravirine, but more evidence is needed to characterize the nature and onset of these interactions. Interactions involving ritonavir-boosted PIs are most frequent when warfarin is initiated in patients receiving concurrent efavirenz therapy. It seems prudent to base warfarin dosage adjustments on International Normalized Ratio (INR) response rather than empirically beginning with a lower warfarin dose. Similarly, INR response should be used to guide warfarin dosage requirements in patients on nevirapine, nelfinavir, lopinavir/ritonavir, or other ritonavir-boosted PIs rather than starting with higher baseline doses.^114^–^117^

Health care providers should be alert to the potential for dangerously high or low INRs when they are giving anticoagulants to patients with HIV infection who are undergoing antiretroviral therapy. Perhaps alternative forms of anticoagulation, such as low-molecular-weight heparin (LMWH), should be considered in some cases in the HIV-infected population receiving antiretroviral therapy, given the absence of compliance in some HIV-affected subjects. LMWH should be a safer choice in those patients, always keeping in mind that HIV infection may be an independent risk factor for the development of heparin-induced thrombocytopenia (HIT).^118^ Lastly the timing of administration of warfarin and certain antiretroviral agents should be staggered.

## Conclusions:

**Figure 2:**
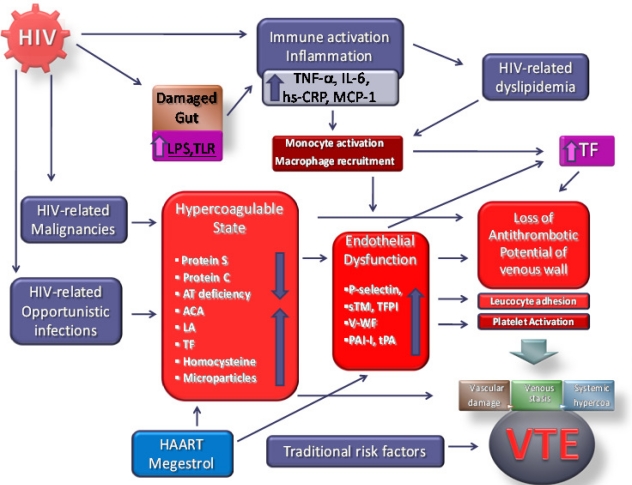
Diagram summarizing the pathogenesis of HIV-related VTE. HIV, human immunodeficiency virus; LPS, lipopolysaccharide; TLR, toll-like receptor; AT, antithrombin; ACA, anticardiolipin antibodies; LA, lupus anticoagulant; TF, Tissue Factor; TNF-a, tumor necrosis factor alpha; IL-6, interleukin six; hsCRP, high-sensitivity C-reactive protein; MCP1, monocyte chemotactic protein-1; sTM, soluble thrombomodulin; TFPI, tissue factor pathway inhibitor; v-WF, von Willebrand Factor; PAI-I, plasminogen activator inhibitor-1; tPA, tissue plasminogen activator

Currently available epidemiological evidence suggests that chronic HIV infection is associated with a two to tenfold increased risk of venous thrombosis in comparison with a general population of the same age. Some risk factors demonstrated a strongest association with VTE such as like a diagnosis of AIDS, low CD4^+^ cell count especially in the presence of clinical AIDS, protein S deficiency, and protein C deficiency. (Figure 2) Whereas other risk factors are still controversial like protease inhibitor therapy, presence of active opportunistic infections and presence of antiphospholipid antibodies, including anticardiolipin antibodies and lupus anticoagulant. With this understanding, appropriate prophylactic measures can be instituted, which may include universal VTE prophylaxis with oral anticoagulants or low-molecular weight heparin on hospitalization and for high-risk outpatients. Physicians caring for HIV positive patients should be able to recognize and treat not only the well-known opportunistic infections and malignancies associated with this chronic disease, but also be alert to the less well-known complications such as thromboses. Pulmonary embolism should be included in the differential diagnosis when patients with HIV/AIDS have unexplained dyspnea or hypoxemia. In younger individuals with VTE, especially men, without other identifiable risk factors for VTE, HIV should be considered.

Finally new well-designed case control studies are needed to answer several open questions: Do traditional factors associated with increased VTE risk have the same impact in HIV patients? Does HAART contribute to VTE risk in HIV and How? Are traditional screening methods applicable in HIV? Are traditional VTE risk management strategies applicable in HIV?

## Figures and Tables

**Table 1:** Main studies on VTE incidence in HIV patients.

**Author**	**Years studied**	**Population Size**	**VTE Incidence**
**Jenkins^8^**	1991	243	3.29 %
**Laing^9^**	1996	728	0,96%
**Howling^10^**	1999	3792	1,07%
**George^11^**	1999	650	0,19–1,07%
**Sullivan^12^**	2000	42935	0,26%
**Saber^13^**	2001	4752	0,95%
**Saif^14^**	2001	131	7,63%
**Copur^15^**	2002	362	2,76%
**Fulz 16**	2004	13549-514	2,0%-1,6%
**Ahonkhai^17^**	1989–2004	160	0,54%
**Malek 18**	1996–2004	6944	0.52
**Crum-Cianflone^19^**	1996–2007	465	3,7%
**Rasmussen^20^**	1995–2007	4333	8.0%–1,5%; norm pop 0,3%

## References

[1] Global report: UNAIDS report on the global AIDS epidemic 2010. www.unaids.org (accessed 16 May 2011)

[2] Palella FJ, Delaney KM, Moorman AC, Loveless MO, Fuhrer J, Satten GA, Aschman DJ, Holmberg SD (1998). Declining morbidity and mortality among patients with advanced human immunodeficiency virus infection. HIV Outpatient Study Investigators. N. Engl. J. Med.

[3] Murphy EL, Collier AC, Kalish LA, Assmann SF, Para MF, Flanigan TP, Kumar PN, Mintz L, Wallach FR, Nemo GJ (2001). Highly active antiretroviral therapy decreases mortality and morbidity in patients with advanced HIV disease. Ann Inter Med.

[4] Mocroft A, Reiss P, Gasiorowski J, Ledergerber B, Kowalska J, Chiesi A, Gatell J, Rakhmanova A, Johnson M, Kirk O, Lundgren J (2010). Serious fatal and non fatal non AIDS defining illnesses in Europe. J Acquir Immune Defic Syndr.

[5] Naess IA, Christiansen SC, Romundstad P, Cannegieter SC, Rosendaal FR, Hammerstrøm J (2007). Incidence and mortality of venous thromboembolism: a population based study. J Thromb Haemost.

[6] Cohen AT, Tapson VF, Bergmann JF, Goldhaber SZ, Kakkar AK, Deslandes B, Huang W, Zayaruzny M, Emery L, Anderson FA (2008). Venous thromboembolism risk and prophylaxis in the acute hospital care setting (ENDORSE study): a multinational cross-sectional study. Lancet.

[7] Hirsh J, Guyatt G, Albers GW, Harrington R, Schünemann HJ (2008). Antithrombotic and thrombolytic therapy: American College of Chest Physicians Evidence-Based Clinical Practice Guidelines (8th Edition). Chest.

[8] Jenkins RE, Peters BS, Pinching AJ (1991). Thromboembolic disease in AIDS is associated with cytomegalovirus disease. AIDS.

[9] Laing RBS, Brettle RP, Leen CLS (1996). Venous thrombosis in HIV infection. Int J STD AIDS.

[10] Howling SJ, Shaw PJ, Miller RF (1999). Acute pulmonary embolism in patients with HIV disease. Sex Transm Infect.

[11] George SL, Swindells S, Knudson R, Stapleton JT (1999). Unexplained Thrombosis in HIV-infected Patients Receiving Protease Inhibitors: Report of Seven Cases. Am J Med.

[12] Sullivan PS, Dworkin MS, Jones JL, Hooper WG (2000). Epidemiology of thrombosis in HIV-infected individuals. AIDS.

[13] Saber AA, Aboolian A, LaRaja RD, Baron H, Manna K (2001). HIV/AIDS and the risk of deep vein thrombosis a study of 45 patients with lower extremity involvement. Am Surg.

[14] Saif MW, Bona R, Greenberg B (2001). AIDS and thrombosis: retrospective study of 131 HIV-infected patients. AIDS Patient Care STDS.

[15] Copur AS, Smith PR, Gomez V, Bergman M, Homel P (2002). HIV infection is a risk factor for venous thromboembolism. AIDS Patient Care STDS.

[16] Fultz SL, McGinnis KA, Skanderson M, Ragni MV, Justice AC (2004). Association of venous thromboembolism with human immunodeficiency virus and mortality in veterans. Am J Med.

[17] Ahonkhai AA, Gebo KA, Streiff MB, Moore RD, Segal JB (2008). Venous thromboembolism in patients with HIV/AIDS: a case control study. J Acquir Immune Defic Syndr.

[18] Malek J, Rogers R, Kufera J, Hirson JM (2011). Venous Thromboembolic disease in the HIV-infected patient. Am J Emerg Med.

[19] Crum-Cianflone NF, Weekes J, Bavaro M (2008). Thromboses among HIV-infected patients during the highly active antiretroviral therapy era. AIDS Patient Care STDS.

[20] Rasmussen LD, Dybdal M, Gerstoft J, Kronborg G, Larsen CS, Pedersen C, Pedersen G, Jensen J, Pedersen L, Sørensen HT, Obel N (2011). HIV and risk of venous thromboembolism: a Danish nationwide population-based cohort study. HIV Med.

[21] Goldhaber SZ (2010). Risk Factors for Thromboembolism. JACC.

[22] Wilkerson WR, Sane DC Aging and Thrombosis. Semin Thromb and haemost 2002.

[23] Jacobson MC, Debuze BJ, Aboulafia DM (2004). Thrombotic complications in patients infected with HIV in the era of Highly active antiretroviral therapy: a case series. Clinc Infect Dis.

[24] Appay V, Rowland-jones SL (2002). Premature ageing of the immune system: the cause of AIDS. Trends Immunol.

[25] Deeks SG, Phillips AN HIV infection, antiretroviral treatment, ageing, and non-AIDS related morbidity. BJM.

[26] Kuller LH, Tracy R, Belloso W, De Wit S, Drummond F, Lane HC, Ledergerber B, Lundgren J, Neuhaus J, Nixon D, Paton NI, Neaton JD (2008). Inflammatory and coagulation biomarkers and mortality in patients with HIV infection. PLoS Med.

[27] Lillam AL (1993). Cardiovascular and Thrombosis pathology associated with cocaine use. Hematol Oncol Clin North Am.

[28] Syed FF, Beeching NJ (2005). Lower-limb deep-vein thrombosis in a general hospital:risk factors, outcome and the contribution of intravenous drug use. QJM.

[29] Lafeuillade A, Alessi MC, Poizot-Martin I, Dhiver C, Quilichini R, Aubert L, Gastaut JA, Juhan-Vague I (1991). Protein S deficiency and HIV infection. N Engl J Med.

[30] Lafeuillade A, Alessi MC, Poizot-Martin I, Boyer-Neumann C, Zandotti C, Quilichini R, Aubert L, Tamalet C, Juhan-Vague I, Gastaut JA (1992). Endothelial cell dysfunction in HIV infection. J Acquir Immune Defic Syndr.

[31] Majluf-Cruz A, Silva-Estrada M, Sánchez-Barboza R, Montiel-Manzano G, Trevino-Pérez S, Santoscoy-Gómez M, de Chávez-Ochoa AR, Corona-de la Pena N, Nieto-Cisneros L (2004). Venous Thrombosis among patients with AIDS. Clin Appl Thromb Hemost.

[32] Lijfering WM, Ten Kate MK, Sprenger HG, Van der Meer J (2006). Absolute risk of venous and arterial thrombosis in HIV-infected patients and effects of combination antiretroviral therapy. J Thromb Haemost.

[33] Anderson JAM, Weitz JI (2010). Hypercoagulable States. Clin Chest Med.

[34] Sorice M, Griggi T, Arcieri P, Circella A, d'Agostino F, Ranieri M, Modrzewska R, Lenti L, Mariani G (1994). The role of Anticardiolipin and anti-Protein S Antibodies. Thromb Res.

[35] Hooper WC, Phillips DJ, Ribeiro MJ, Benson JM, George VG, Ades EW, Evatt BL (1994). Tumor Necrosis Factor-alfa downregulates Protein S secretion in Human Microvascular and Umbelical Vein Endothelial Cells but not in the HepG-2 Hepatoma Cell Line. Blood.

[36] De Larranaga GF, Forastiero RR, Carreras LO, Alonso BS (1999). Different types of antiphospholipid antibodies in AIDS.: A comparison with syphilis and the antiphospholipid syndrome. Thromb Res.

[37] Feffer SE, Fox RL, Orsen MM, Harajai KJ, Glatt AE (1995). Thrombotic tendencies and correlation with clinical status in patients infected with HIV. South Med J.

[38] Demers C, Ginsberg JS, Hirsh J, Henderson P, Blajchman MA (1992). Thrombosis in AT III deficiency persons. Report of a large kindred and literature review. Ann Inter MeD.

[39] Flinn WR, McDaniel MD, Yao JS (1984). Antithrombin III deficiency as a reflection of dynamic protein metabolism in patients undergoing vascular reconstruction. J Vasc Surgery.

[40] Espinosa G, Cervera R (2010). Antiphospholipid syndrome: frequency, main cause and risk factors of mortality. Nat Rev Rheaumatol.

[41] Sene D, Piette JC, Cacoub P (2008). Antiphospholipid antibodies, antiphospholipid syndrome and infections. Autoimmune Rev.

[42] Galrão L, Brites C, Atta ML, Atta A, Lima I, Gonzalez F, Magalhães F, Santiago M (2007). Antiphospholipid antibodies in HIV-positive patients. Clin Rheumatol.

[43] Palomo I, Alarcón M, Sepulveda C, Pereira J, Espinola R, Pierangeli S (2003). Prevalence of antiphospholipid and antiplatelet antibodies in human immnunodeficiency virus(HIV)-infected Chilean Patients. J Clin Lab Anal.

[44] Leder AN, Flansbaum B, Zandman-Goddard G, Asherson R, Shoenfeld Y (2001). Antiphospolipid syndrome induced by HIV. Lupus.

[45] Uthman IW, Gharavi AE (2002). Viral infections and antiphospholipid antibodies. Semin Arthritis Rheum.

[46] Shahnaz S, Parikh G, Opran A (2004). Antiphospholipid antibody syndrome manifesting as a deep venous thrombosis and pulmonary embolism in a patient with HIV. Am J Med Sci.

[47] Funderburg NT, Mayne E, Sieg SF, Asaad R, Jiang W, Kalinowska M, Luciano AA, Stevens W, Rodriguez B, Brenchley JM, Douek DC, Lederman MM (2010). Increased tissue factor expression on circulating monocytes in chronic HIV infection: relationship to in vivo coagulation and immune activation. BLOOD.

[48] Garcia F, Fumero E, Gatell JM (2008). Immune modulators and treatment interruption. Curr Opin HIV/AIDS.

[49] Morel O, Toti F, Hugel B, Bakouboula B, Camoin-Jau L, Dignat-George F, Freyssinet JM (2006). Procoagulant microparticles: disrupting the vascular homeostasis equation. Arterioscler ThrombVascBiol.

[50] Mackman N, Tilley RE, Key Ns (2007). Role of the extrinsic pathway of blood coagulation in hemostasis and thrombosis. Arterioscler ThrombVascBiol.

[51] Eyal A, Velelr M (2009). HIV and venous thrombotic events. S Afr J Surg.

[52] Bernasconi E, Uhr M, Magenta L, Ranno A, Telenti A (2001). Homocysteinemia in HIV-infected patients treated with highly active antiretroviral therapy. AIDS.

[53] Guaraldi G, Ventura P, Garlassi E, Orlando G, Squillace N, Nardini G, Stentarelli C, Zona S, Marchini S, Moriondo V, Tebas P (2009). Hyperhomocysteinemia in HIV-infected patients: determinants of variability and correlations with predictors of cardiovascular disease. HIV Medicine.

[54] Eichinger S, Stümpflen A, Hirschl M, Bialonczyk C, Herkner K, Stain M, Schneider B, Pabinger I, Lechner K, Kyrle PA (1998). Hyperhomocysteinemia is a risk factor of recurrent venous thromboembolism. Thromb Haemost.

[55] den Heijer M, Willems HP, Blom HJ, Gerrits WB, Cattaneo M, Eichinger S, Rosendaal FR, Bos GM (2007). Homocysteine lowering by B vitamins and the secondary prevention of deep vein thrombosis and pulmonary embolism: a randomized, placebo-controlled, double-blind trial. Blood.

[56] Kyrle PA, Rosendaal FR, Eichinger S (2010). Risk assessment for recurrent venous thrombosis. Lancet.

[57] Gresele P, Momi S, Migliacci R (2010). Endothelium, venous thromboembolism and ischemic cardiovascular events. Thromb Haemost.

[58] Migliacci R, Becattini C, Pesavento R, Davi G, Vedovati MC, Guglielmini G, Falcinelli E, Ciabattoni G, Dalla Valle F, Prandoni P, Agnelli G, Gresele P (2007). Endothelial dysfunction in patients with spontaneous venous thromboembolism. Haematologica.

[59] Gresele P, Migliacci R, Vedovati MC, Ruffatti A, Becattini C, Facco M, Guglielmini G, Boscaro E, Mezzasoma AM, Momi S, Pengo V (2009). Patients with primary antiphospholipid antibody syndrome and without associated vascular risk factors present a normal endothelial function. Thromb Res.

[60] Wakefield TW, Myers DD, Henke PK (2008). Mechanism of venous thrombosis and resolution. Arterioscler Thromb Vasc Biol.

[61] Pomerantz RJ, Kuritzkes DR, de la Monte SM, Rota TR, Baker AS, Albert D, Bor DH, Feldman EL, Schooley RT, Hirsch MS (1987). Infection of the retina by human immunodeficiency virus type I. N Engl J Med.

[62] Solages A, Vita JA, Thornton DJ, Murray J, Heeren T, Craven DE, Horsburgh CR (2006). Endothelial function in HIV-infected persons. Clin Infect Dis.

[63] Chi D, Henry J, Kelley J, Thorpe R, Smith JK, Krishnaswamy G (2000). The effects of HIV infection on endothelial function. Endothelium.

[64] Musselwhite LW, Sheikh V, Norton TD, Rupert A, Porter BO, Penzak SR, Skinner J, Mican JM, Hadigan C, Sereti I (2011). Markers of endothelial dysfunction, coagulation and tissue fibrosis independently predict venous thromboembolism in HIV. AIDS.

[65] Polgar J, Matuskova J, Wagner DD (2005). The P-selectin, tissue factor,coagulation triad. J Thromb Haemost.

[66] Rectenwald JE, Myers DD, Hawley AE, Longo C, Henke PK, Guire KE, Schmaier AH, Wakefield TW (2005). D-dimer, P-selectin, and microparticles: novel markers to predict deep venous thrombosis. A pilot study. Thromb Haemost.

[67] Ross AC, Armentrout R, O'Riordan MA, Storer N, Rizk N, Harrill D, El Bejjani D, McComsey GA (2008). Endothelial activation markers are linked to HIV status and are independent of antiretroviral therapy and Lipoatrophy. J Acquir Immune Defic Syndr.

[68] Schved JF, Gris JC, Arnaud A, Martinez P, Sanchez N, Wautier JL, Sarlat C (1992). von Willebrand factor antigen, tissue-type plasminogen activator antigen, and risk of death in human immunodeficiency virus 1-related clinical disease: independent prognostic relevance of tissue-type plasminogen activator. J Lab Clin Med.

[69] Toulon P, Lamine M, Ledjev I, Guez T, Holleman ME, Sereni D, Sicard D (1993). Heparin Cofactor II deficiency in patients infected with the Human Immunodeficiency Virus. Thromb Haemost.

[70] Rolinski B, Geier SA, Sadri I, Klauss V, Bogner JR, Ehrenreich H, Goebel FD (1994). Endothelin-1 immunoreactivity in plasma is elevated in HIV-1 infected patients with retinal microangiopathic syndrome. Clin Invest.

[71] Seigneur M, Constans J, Blann A, Renard M, Pellegrin JL, Amiral J, Boisseau M, Conri C (1997). Soluble adhesion molecules and endothelial cell damage in HIV infected patients. Thromb Haemost.

[72] Hadigan C, Meigs JB, Rabe J, D'Agostino RB, Wilson PW, Lipinska I, Tofler GH, Grinspoon SS (2001). Increased PAI-1 and tPA antigen levels are reduced with metformin therapy in HIV-infected patients with fat redistribution and insulin resistance. J Clin Endocrinol Metab.

[73] Schecter AD, Berman AB, Yi L, Mosoian A, McManus CM, Berman JW, Klotman ME, Taubman MB (2001). HIV envelope gp120 activates human arterial smooth muscle cells. Proc Natl Acad Sci USA.

[74] Sugerman RW, Church JA, Goldsmith JC, Ens GE (1996). Acquired protein S deficiency in children infected with human immunodeficiency virus. Pediatr Infect Dis J.

[75] Klein SK, Slim EJ, de Kruif MD, Keller TT, ten Cate H, van Gorp EC, Brandjes DP (2005). Is chronic HIV infection associated with venous thrombotic disease? A systematic review. Neth J Med.

[76] Karpatkin S, Nardi M, Green D (2002). Platelet and coagulation defects associated with HIV-1-infection. Thromb Haemost.

[77] Pinilla J, Hill AR (1992). Thromboembolism associated with acquired immunodeficiency syndrome [letter]. Chest.

[78] Kiser LK, Badowski ME (2010). Risk factors for venous thromboembolism in patients with Human Immunodeficiency Virus Infection. Pharmacotheraphy.

[79] Baker JV, Neuhaus J, Duprez D (2011). Changes in Inflammatory and Coagulation Biomarkers: A Randomized Comparison of Immediate versus Deferred Antiretroviral Therapy in Patients With HIV Infection. J Acquir Immune Defic Syndr.

[80] Abgueguen P, Delbos V, Chennebault JM, Payan C, Pichard E (2003). Vascular thrombosis and acute cytomegalovirus infection in immunocompetent patients: Report of 2 cases and literature review. Clin Inf Dis.

[81] Lijfering WM, Sprenger HG, Son van WJ, Van Der Meer J (2007). Mesenteric vein thrombosis associated with primary cytomegalovirus infection: A case report. Blood Coagul Fibrinolysis.

[82] Abgueguen P, Delbos V, Ducancelle A, Jomaa S, Fanello S, Pichard E (2010). Venous thrombosis in immunocompetent patients with acute cytomegalovirus infection: A complication that may be underestimated. Clin Microbiol Infect.

[83] Tichelaar VY, Sprenger HG, Mäkelburg AB (2011). Active cytomegalovirus infection in patients with acute venous thrombosis: A case-control study. Am J Hematol.

[84] Justo D, Finn T, Atzmony L, Guy N, Steinvil A (2011). Thrombosis associated with acute cytomegalovirus infection: a meta-analysis. Eur J Intern Med.

[85] Atzmony L, Halutz O, Avidor B, Finn T, Zimmerman O, Steinvil A, Zeltser D, Giladi M, Justo D (2010). Incidence of cytomegalovirus-associated thrombosis and its risk factors: a case-control study. Thromb Res.

[86] Centers for Disease Control and Prevention (2009). Guidelines for prevention and treatment of opportunistic infections in HIV infected adults and adolescents. MMWR Recomm Rep.

[87] Mulder R, Tichelaar YI, Sprenger HG, Mulder AB, Lijfering WM (2010). Relationship between cytomegalovirus infection and procoagulant changes in human immunodeficiency virus-infected patients. Clin Microbiol Infect.

[88] Aboulafia DM, Bundow D, Waide S, Bennet C, Kerr D (2000). Initial observations on the efficacy of highly active antiretroviral therapy in the treatment of HIV-associated autoimmune thrombocytopenia. Am J Med Sci.

[89] Rosen MJ (2008). Respirology.: Pulmonary complications of HIV infection.

[90] Santiago MB, Cossermelli W, Tuma MF, Pinto MN, Oliveira RM (1989). Anticardiolipin antibodies in patients with infectious diseases. Clin. Rheumat.

[91] Cohen AJ, Philips TM, Kessler CM (1986). Circulating coagulation inhibitors in the acquired immunodeficiency syndrome. Ann Intern Med.

[92] Turken O, Kunter E, Sezer M, Solmazgul E, Cerrahoglu K, Bozkanat E, Ozturk A, Ilvan A (2002). Haemostatic changes in active pulmonary tuberculosis. Int J Tuberc Lung Dis.

[93] White NW (1989). Venous thrombosis and rifampicin. Lancet.

[94] Ambrosetti M, Ferrarese M, Codecasa LR, Besozzi G, Sarassi A, Viggiani P, Migliori GB (2006). Incidence of Venous Thromboembolism in Tuberculosis Patients. Respiration.

[95] Khorana AA, Connolly GC (2009). Assessing Risk of Venous Thromboembolism in the Patient With Cancer. J Clin Oncol.

[96] Scates SM (1992). Diagnosis and treatment of cancer- related thrombosis. Hematol Oncol Clin North Am.

[97] Engels EA, Pfeiffer RM, Goedert JJ, Virgo P, McNeel TS, Scoppa SM, Biggar RJ (2006). Trends in cancer risk among people with AIDS in the United States 1980–2002. AIDS.

[98] Engels EA, Biggar RJ, Hall HI, Cross H, Crutchfield A, Finch JL, Grigg R, Hylton T, Pawlish KS, McNeel TS, Goedert JJ (2008). Cancer risk in people infected with human immunodeficiency virus in the United States. Int J Cancer.

[99] Schneider E, Whitmore S, Glynn KM, Dominguez K, Mitsch A, McKenna MT (2008). Revised surveillance case definitions for HIV infection among adults, adolescents, and children aged <18 months and for HIV infection and AIDS among children aged 18 months to <13 years–United States, 2008. MMWR Recomm Rep.

[100] Shiels MS, Pfeiffer RM, Gail MH, Hall HI, Li J, Chaturvedi AK, Bhatia K, Uldrick TS, Yarchoan R, Goedert JJ, Engels EA (2011). Cancer Burden in the HIV-Infected Population in the United States.. J Natl Cancer Inst.

[101] Kaufmann T, Nisce LZ, Metroka C (1991). Thromboembolism in AIDS-related Kaposi's sarcoma. JAMA.

[102] Barbaro G (2002). Cardiovascular manifestations of HIV infection. Circulation.

[103] Lyonne L, Magimel C, Cormerais L, Trouillier S, Bocquier B, Zenut M, Jacomet C, Laurichesse H, Beytout J, Lesens O (2008). Thromboembolic events at the time of highly active antiretroviral therapies against human immunodeficiency virus]. Rev Med Interne.

[104] Shen YM, Frenkel EP (2004). Thrombosis and a hypercoagulable state in HIV-infected patients. Clin Appl Thromb Hemost.

[105] Fernández Sánchez C, Marín Gámez N, López Martínez G, Carbayo Gorriz C (1998). Thrombophlebitis by megestrol acetate in patients with human immunodeficiency virus infection. Med Clin (Barc).

[106] Force L, Barrufet P, Herreras Z, Bolibar I (1999). Deep venous thrombosis and megestrol in patients with HIV infection. AIDS.

[107] Nagaraja V, Terriquez JA, Gavini H, Jha L, Klotz SA (2010). Pulmonary Embolism Mimicking pneumonia in HIV patient. 2010. Case Report Med.

[108] Narayanan TS, Narawane NM, Phadke AY, Abraham P (1998). Multiple abdominal venous thrombosis in HIV-seropositive patient. Indian J Gastroenterol.

[109] Soentjens P, Ostyn B, Van Outryve S, Ysebaert D, Vekemans M, Colebunders R (2006). Portal vein thrombosis in a patient with HIV treated with a protease inhibitor-containing regimen. Acta Clin Belg.

[110] Heit JA, Kobbervig CE, James AH, Petterson TM, Bailey KR, Melton LJ (2005). Trends in the incidence of venous thromboembolism during pregnancy or postpartum: a 30-year population-based study. Ann Intern Med.

[111] Pham PA, Flexner C (2011). Emerging antiretroviral drug interactions. J Antimicrob Chemother.

[112] Holbrook AM, Pereira JA, Labiris R, McDonald H, Douketis JD, Crowther M, Wells PS (2005). Systematic overview of warfarin and its drug and food interactions. Arch Intern Med.

[113] Fulco PP, Zingone MM, Higginson RT (2008). Possible antiretroviral therapy–warfarin drug interaction. Pharmacotherapy.

[114] Bonora S, Lanzafame M, D'Avolio A, Trentini L, Lattuada E, Concia E, Di Perri G (2008). Drug interactions between warfarin and efavirenz or lopinavir-ritonavir in clinical treatment. Clin Infect Dis.

[115] Gatti G, Alessandrini A, Camera M, Di Biagio A, Bassetti M, Rizzo F (1998). Influence of indinavir and ritonavir on warfarin anticoagulant activity. AIDS.

[116] Knoell KR, Young TM, Cousins ES (1998). Potential interaction involving warfarin and ritonavir. Ann Pharmacother.

[117] Hughes CA, Freitas A, Miedzinski LJ (2007). Interaction between lopinavir/ritonavir and warfarin. CMAJ.

[118] Thompson GR, Lawrence VA, Crawford GE (2007). HIV infection increases the risk of heparin-induced thrombocytopenia. Clin Infect Dis.

